# Concordance and Discordance Rates of V-Raf Murine Sarcoma Viral Oncogene Homolog B1 (*BRAF*)^V600E^ Status in Metastatic against Primary Lesion of Melanoma: A Meta-analysis

**DOI:** 10.31662/jmaj.2020-0016

**Published:** 2020-07-07

**Authors:** Hiroshi Yokomichi, Takashi Inozume, Makoto Wada, Jun Asai, Hiroshi Igaki, Kenjiro Namikawa, Ayato Hayashi, Satoshi Fukushima, Taku Fujimura, Hiroshi Koga, Yasuhiro Nakamura, Mie Mochizuki, Zentaro Yamagata

**Affiliations:** 1 Department of Health Sciences, University of Yamanashi, Chuo, Japan; 2 Department of Dermatology, University of Yamanashi, Chuo, Japan; 3 Department of Dermatology, Kyoto Prefectural University of Medicine, Kyoto, Japan; 4 Department of Radiation Oncology, National Cancer Center Hospital, Tokyo, Japan; 5 Department of Dermatologic Oncology, National Cancer Center Hospital, Tokyo, Japan; 6 Department of Plastic and Reconstructive Surgery, Juntendo University Urayasu Hospital, Urayasu, Japan; 7 Department of Dermatology and Plastic Surgery, Faculty of Life Sciences, Kumamoto University, Kumamoto, Japan; 8 Department of Dermatology, Tohoku University, Sendai, Japan; 9 Department of Dermatology, Shinshu University School of Medicine, Matsumoto, Japan; 10 Department of Skin Oncology/Dermatology, Saitama Medical University International Medical Center, Hidaka, Japan; 11 Department of Pediatrics, University of Yamanashi, Chuo, Japan

**Keywords:** melanoma, *BRAF*^V600E^, primary tumor, metastasis, BRAF mutation, disagreement, serine-threonine protein kinase BRAF inhibitor, mitogen-activated protein kinase kinase inhibitor

## Background

New effective molecular-targeted agents ^(1)^ including combination serine-threonine protein kinase BRAF inhibitor and mitogen-activated protein kinase kinase (MEK) inhibitor (BRAF/MEK inhibitor) therapy have improved overall survival (OS) and progression-free survival (PFS) of patients with metastatic melanoma with *BRAF*^V600E/K^ mutations ^(2), (3), (4)^. Observational studies suggest that 40%-60% Caucasians and < 30% Japanese are *BRAF*^V600E/K^ mutation positive ^(5)^. BRAF/MEK inhibitors are only approved for *BRAF*^V600E/K^ mutation-positive patients because they are only effective against lesions with this mutation ^(6)^.

The mutational status of metastatic lesions cannot easily be tested because melanoma metastasizes to subcutaneous and superficial lymph nodes, visceral areas, and deep lymph nodes; therefore, in such cases, whether BRAF/MEK inhibitors are suitable for patients with melanoma is unclear ^(7)^. In clinical practice, primary skin lesions resected during primary treatment are genetically tested to predict the mutational status of inaccessible metastatic lesions.

Some cases might have *BRAF*^V600E/K^ mutation-positive primary lesions, but have negative metastatic lesions, or vice versa. In the former, BRAF/MEK inhibitors would not be effective, whereas in the latter, the patient loses the opportunity to receive effective treatment. Disagreement in the proportion of *BRAF* mutations between primary and metastatic lesions varies ^(8)^. A previous study did not address the probability of agreement for metastasis when the primary lesion was *BRAF* mutation positive or *BRAF* mutation negative. Moreover, disagreement proportions for all *BRAF* mutations were included, although molecular-targeted therapy is currently approved only for patients with *BRAF*^V600E/K^ ^(8)^.

Calculating the mutation probability in metastatic lesions when the primary lesion mutation is known allows the more appropriate use of BRAF/MEK inhibitors. The probability of *BRAF*^V600E^-positive [*BRAF*(+)] metastatic lesions when the primary cancer lesion was *BRAF*(+) and *BRAF*^V600E^-negative [*BRAF*(−)] metastatic lesions when the primary cancer lesion was *BRAF*(−) was calculated.

## Methods

### Search methodology and inclusion/exclusion criteria

We searched the PubMed, Cochrane Library, and Igaku Chuo Zasshi databases for observational studies on November 23, 2017. Search criteria with MeSH terms were published elsewhere ^(9)^. One investigator selected articles that potentially met the criteria on the basis of their titles and abstracts. For eligible studies, the same investigator abstracted the data independently using a predefined form. Searched studies used real-time PCR, Sanger sequencing, and *BRAF*^V600E^-specific immunohistochemistry to detect mutants. Therefore, we set the outcome of *BRAF*^V600E^ mutations as those detected by the searched methods and that were predominant in 79%-90% of patients ^(10), (11)^. We included studies investigating *BRAF*^V600E^ status between primary melanoma and metastatic lesions in the same patients, irrespective of testing methods, and those without *BRAF*^V600E^ testing were excluded.

### Statistical analysis

We integrated the probabilities of *BRAF*^V600E^ positive and negative in metastatic lesions when primary lesions were* BRAF*^V600E^**positive and negative, respectively: *BRAF*(+) metastatic lesion given the primary lesion**is *BRAF*(+), and *BRAF*(−) metastatic lesion given the primary lesion**is *BRAF*(−). To determine these probabilities, we used primary/metastatic lesion [mutation positive/negative, represented as *BRAF*(+) or (−)/*BRAF*(+) or (−)] information for each individual. We calculated the integrated probability (95% confidence interval [95%CI]) of *BRAF*(+) metastatic lesions given *BRAF*(+) primary lesions and that of *BRAF*(−) metastatic lesions given *BRAF*(−) primary lesions. To unite disagreements in proportions between test results of primary and metastatic lesions, we included more studies. In this analysis, detailed test results for primary and metastatic lesions were not required, but cross tabulations of *BRAF*(+)/(+), *BRAF*(+)/(−), *BRAF*(−)/(+), and *BRAF*(−)/(−) from total sample populations in each observational study were needed. We integrated the proportions of disagreement for the numbers of *BRAF*(+)/(−) or *BRAF*(−)/(+) cases divided by the numbers of *BRAF*(+)/(+), *BRAF*(+)/(−), *BRAF*(−)/(+), and *BRAF*(−)/(−) cases (all combinations of positive/negative results in primary and metastatic lesions). We presented the proportion of disagreement for the number of discrepant cases divided by the number of consistent plus discrepant cases.

To integrate the probabilities and proportions, we used DerSimonian-Laird estimation (random effect model) and fixed-effects models for meta-analysis. Results are shown as forest plots, and publication bias was checked by funnel plots. We used R x64 V.4.4.2 software for statistical analyses and illustrations. All *P*-values are two-sided, and *P* < 0.05 indicated statistical significance.

This study was approved by the ethics committee of the School of Medicine, University of Yamanashi (approval number: 1894), in accordance with the ethical guidelines and regulations of the Declaration of Helsinki.

## Results

**Figure 1.**
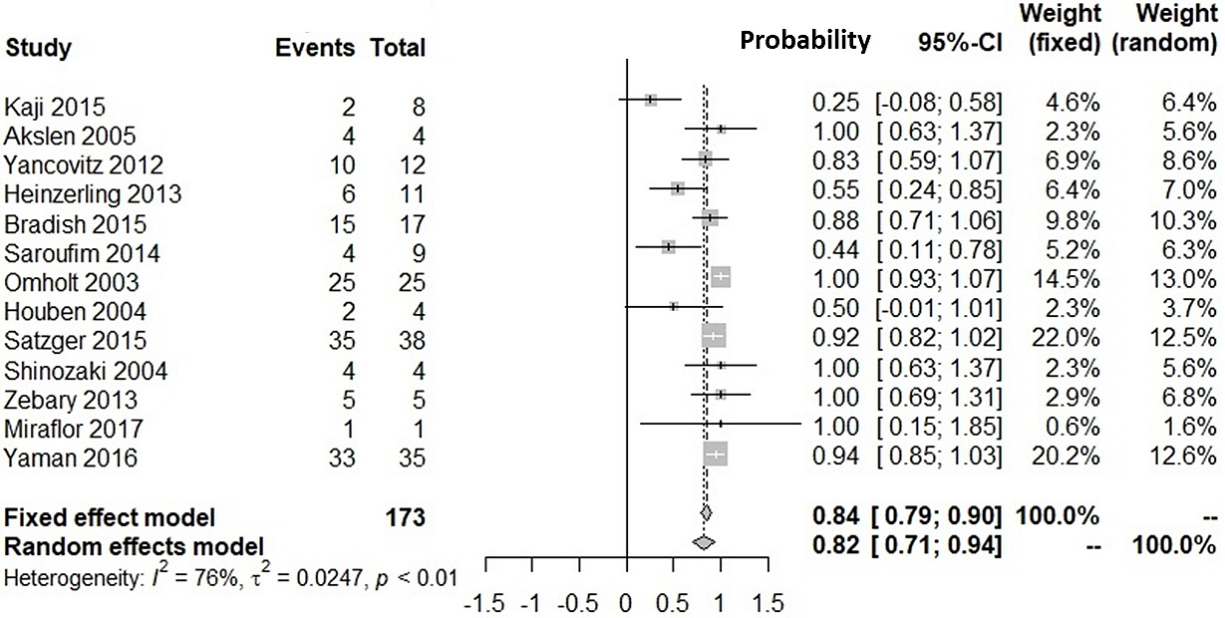
Probability of *BRAF*^V600E^ (+) in metastatic lesions of melanoma against *BRAF*^V600E^ (+) in primary lesions in pairwise comparisons.

**Figure 2.**
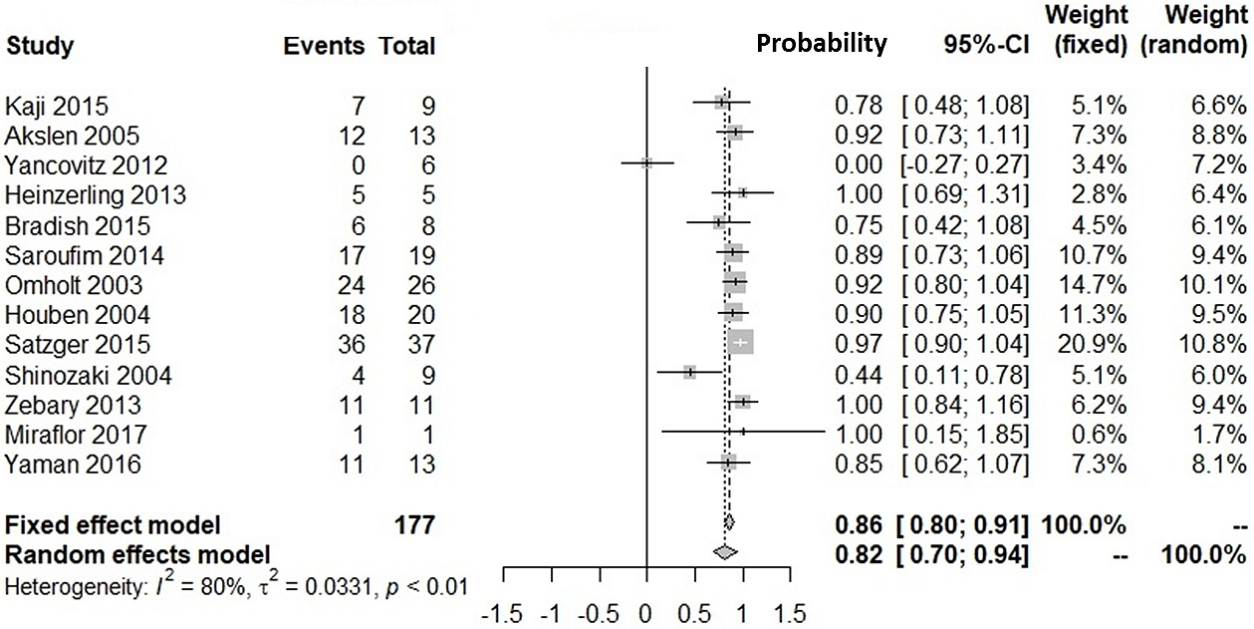
Probability of *BRAF*^V600E^ (−) metastatic lesions of melanoma against *BRAF*^V600E^ (−) in primary lesions in pairwise comparisons.

**Figure 3.**
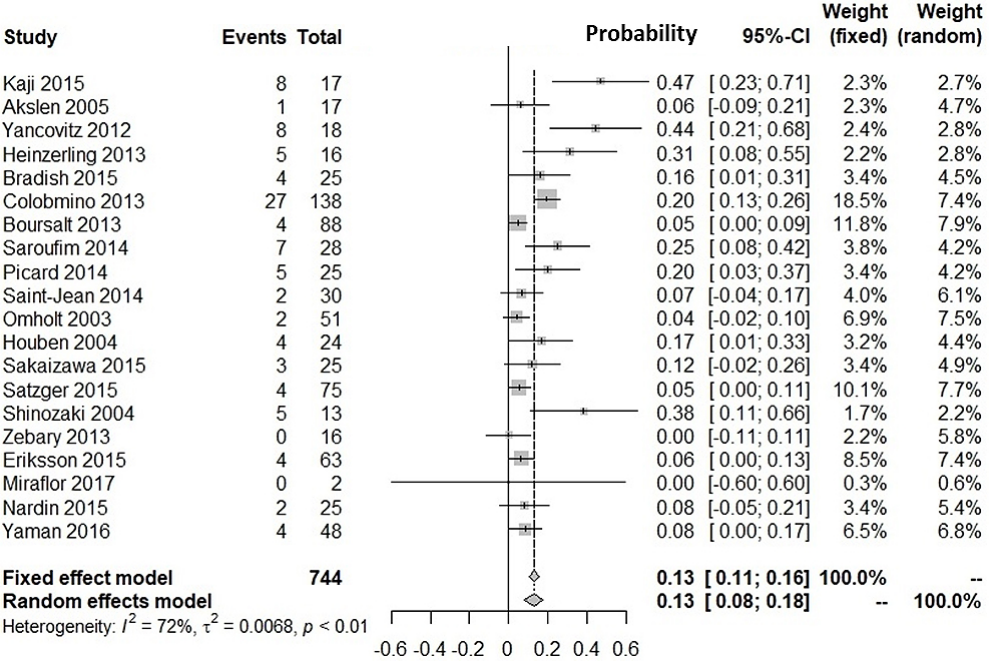
Proportion of disagreement for *BRAF*^V600E^ mutations between primary and metastatic lesions.

Supplementary Figure 1 shows our literature searching and screening processes. Thirteen studies were included in the integration of probabilities of agreement ^(12), (13), (14), (15), (16), (17), (18), (19), (20), (21), (22), (23), (24)^. Figure 1 shows the probabilities of *BRAF*(+) metastatic lesions given *BRAF*(+) primary lesions: the integrated probability is 0.84 (95%CI: 0.79-0.90) in the fixed-effects model and 0.82 (95%CI: 0.71-0.94) in the random-effects model. Figure 2 shows the probabilities of *BRAF*(−) metastatic lesions with *BRAF*(−) primary lesions: the integrated probability is 0.86 (95%CI: 0.80-0.91) in the fixed-effects model and 0.82 (95%CI: 0.70-0.94) in the random-effects model. For the integration of disagreement proportions between primary and metastatic lesions with *BRAF*^V600E^, 20 studies ^(12), (13), (14), (15), (16), (17), (18), (19), (20), (21), (22), (23), (24), (25), (26), (27), (28), (29), (30), (31)^ were included. Figure 3 shows the integrated proportions of 0.13 (95%CI: 0.11-0.16) in the fixed-effects model and 0.13 (95%CI: 0.08-0.18) in the random-effects model. I^2^ indexes for heterogeneity were 76%, 80%, and 72% in Figure 1, Figure 2, and Figure 3, respectively. Funnel plots (Supplementary Figure 2, Figure 3, and Figure 4) indicated no publication bias.

## Discussion

We investigated the probability of *BRAF*^V600E^-positive metastases against *BRAF*^V600E^-positive primary lesions and that of *BRAF*^V600E^-negative metastases against *BRAF*^V600E^-negative primary lesions. Our meta-analyses showed the probability of *BRAF*(+) metastatic lesions with *BRAF*(+) primary lesions was 82% (95%CI: 71%-94%). The probability of *BRAF*(−) metastatic lesions with *BRAF*(−) primary lesions was 82% (95%CI: 70%-94%). The proportion of discrepancies between primary and metastatic lesions was 13% (95%CI: 8%-18%). The proportion was similar with that for epidermal growth factor receptor mutation in patients with non-small cell lung cancer (12.2%) ^(32)^.

BRAF/MEK inhibitors have changed metastatic melanoma treatment. In the beginning, BRAF inhibitor monotherapy has improved OS and PFS in *BRAF*^V600E/K^ patients ^(33)^, ^(34)^. Thereafter, randomized controlled trials showed combination therapy with BRAF/MEK inhibitors was superior to BRAF inhibitor monotherapy regarding OS and PFS ^(2)^. Moreover, a lower frequency of adverse events (rash, alopecia, and skin tumors) was observed for combination therapy compared with that for monotherapy ^(2), (3)^. Thus, combination therapy has become a standard approach ^(7)^.

*BRAF*^V600E/K^ testing in primary lesions is critical for patients with metastasis who may respond to BRAF/MEK inhibitors ^(35)^. Currently, agents are usually administered to patients according to genetic tests performed on primary lesion tissues. Our findings might convince clinicians that metastatic lesions in 82%-87% of patients with *BRAF*^V600E^-positive primary lesions are sensitive to BRAF/MEK inhibitors (Figure 1, Figure 3), whereas 13%-18% would not show an initial response to therapy in clinical trials. Therefore, clinicians might be encouraged to test for *BRAF* mutations in metastatic lesions, because patients with discrepant results between primary and metastatic lesions (14%-18% probability) are often disadvantaged in relation to treatment decisions.

Our study had several strengths. We accumulated the results of 20 worldwide studies, including 15 in a previous meta-analysis of disagreement ^(8)^. We focused on *BRAF*^V600E^, the most frequent and clinically important mutation in melanoma. Funnel plots indicated collated data had no publication bias.

The present study also had some limitations. Selection bias was possible; collated data were from patients who underwent genetic tests, which might decrease the generalizability of the results. Genetic testing methods varied among studies. Ethnic background and stage of melanoma varied and should be considered for treatment decisions after genetic testing. High heterogeneity (I^2^ = 72%-80%) was observed in this meta-analysis. Therefore, the agreement and disagreement proportions might vary among regions.

In conclusion, this meta-analysis revealed 82% of patients had *BRAF*^V600E^-positive metastatic lesions with *BRAF*^V600E^-positive primary lesions and 82% of patients with *BRAF*^V600E^-negative primary lesions had *BRAF*^V600E^-negative metastatic lesions. The proportion of disagreement between *BRAF*^V600E^ mutations in primary and metastatic lesions was 13%. Over-reliance on genetic results of primary tumors might prevent patients with discrepant results receiving appropriate treatment for metastatic lesions. Genetic testing for *BRAF*^V600^ mutations using metastatic tumor samples is suggested, if available, without invasive biopsies.

## Article Information

### Conflicts of Interest

KN received honoraria from Ono Pharmaceutical, Bristol-Myers Squibb, Merck Sharp & Dohme, Novartis Pharmaceutical, Toray Industries, and Takara Bio, outside the submitted work; YN received honoraria from Ono Pharmaceutical, Bristol-Myers Squibb, Maruho, Kyowa Kirin, Merck Sharp & Dohme, Novartis Pharmaceutical, Taisho Toyama Pharma, and Taiho Pharma, outside the submitted work.

### Sources of Funding

This work was partly supported by the Japan Society for the Promotion of Science grant (KAKENHI Grant Number JP18K17376) to HY. The funding source had no role in the design of this study and will not have any role during its execution, analyses, interpretation of the data, or decision to submit results.

### Acknowledgement

We thank Nikki March, PhD, for editing a draft of this manuscript.

### Author Contributions

Conception/design: Inozume T, Yokomichi H

Acquisition of data: All authors

Data analysis: Yokomichi H

Interpretation: All authors

Manuscript writing: Inozume T, Yokomichi H

Final approval of manuscript: All authors

Hiroshi Yokomichi and Takashi Inozume contributed equally to this work.

### Approval by Institutional Review Board (IRB)

This study was approved by the ethics committee of the School of Medicine, University of Yamanashi (approval number: 1894), in accordance with the ethical guidelines and regulations of the Declaration of Helsinki.

### Disclaimer

Zentaro Yamagata is one of the Editors of JMA Journal and on the journal's Editorial Staff. He was not involved in the editorial evaluation or decision to accept this article for publication at all.

## Supplement

Supplementary Figure 1.

Supplementary Figure 2.

Supplementary Figure 3.

Supplementary Figure 4.
