# Supplementary material for: Concordance and Discordance Rates of V-Raf Murine Sarcoma Viral Oncogene Homolog B1 (BRAF)V600E Status in Metastatic against Primary Lesion of Melanoma: A Meta-analysis
Source: JMA J. 2020 Jul 7;3(3):274–9. doi: 10.31662/jmaj.2020-0016 (PMC7590371; doi:10.31662/jmaj.2020-0016)

Supplementary Figure 2. Funnel plot of probability of *BRAF*^{V600E} (+) in metastatic lesion of melanoma against *BRAF*^{V600E} (+) in primary lesion.

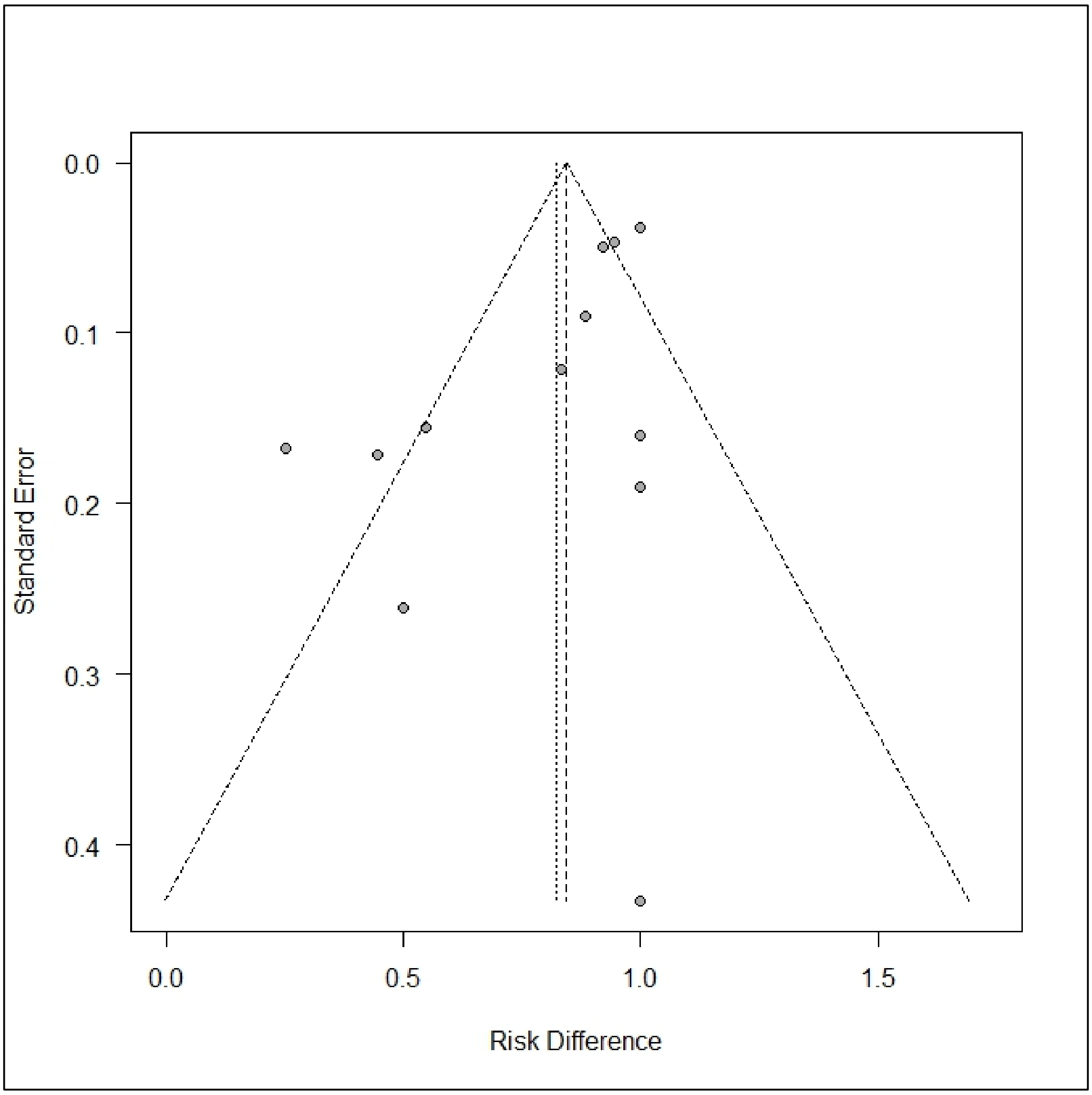

Supplement: Supplementary file 2 — Supplementary Figure 2. [file 2433-3298-3-3-0274-s002.pdf]
