# Supplementary material for: Concordance and Discordance Rates of V-Raf Murine Sarcoma Viral Oncogene Homolog B1 (BRAF)V600E Status in Metastatic against Primary Lesion of Melanoma: A Meta-analysis
Source: JMA J. 2020 Jul 7;3(3):274–9. doi: 10.31662/jmaj.2020-0016 (PMC7590371; doi:10.31662/jmaj.2020-0016)

Supplementary Figure 4. Funnel plot of proportion of discrepancy for *BRAF*^{V600E} mutations between primary and metastatic lesions.

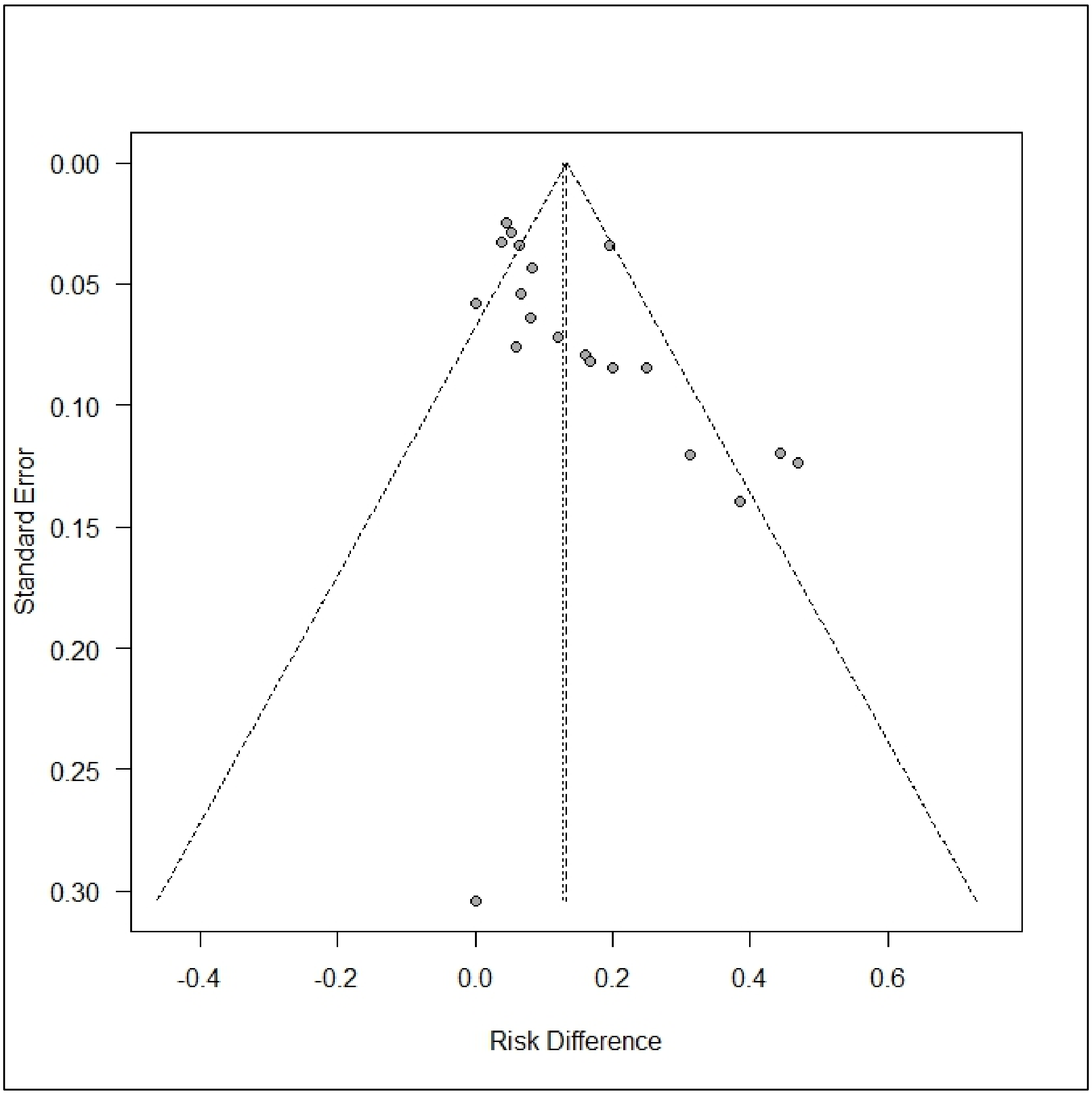

Supplement: Supplementary file 4 — Supplementary Figure 4. [file 2433-3298-3-3-0274-s004.pdf]
